# Extending In-Plane Impedance Measurements from 2D to 3D Cultures: Design Considerations

**DOI:** 10.3390/bioengineering8010011

**Published:** 2021-01-13

**Authors:** Sorel E. De Leon, Lana Cleuren, Zay Yar Oo, Paul R. Stoddart, Sally L. McArthur

**Affiliations:** 1 Bioengineering Research Group, Faculty of Science, Engineering and Technology, Swinburne University of Technology, Hawthorn, VIC 3122, Australia; sdeleonvergara@swin.edu.au (S.E.D.L.); zoo@swin.edu.au (Z.Y.O.); pstoddart@swin.edu.au (P.R.S.); 2 Commonwealth Scientific and Industrial Research Organisation (CSIRO), Clayton, VIC 3168, Australia; 3 PXL University College, Hasselt University, 3500 Hasselt, Belgium; lana.cleuren@gmail.com

**Keywords:** 3D cell culture, electrical impedance spectroscopy, in situ monitoring, cell proliferation, tissue culture

## Abstract

Three-dimensional (3D) cell cultures have recently emerged as tools for biologically modelling the human body. As 3D models make their way into laboratories there is a need to develop characterisation techniques that are sensitive enough to monitor the cells in real time and without the need for chemical labels. Impedance spectroscopy has been shown to address both of these challenges, but there has been little research into the full impedance spectrum and how the different components of the system affect the impedance signal. Here we investigate the impedance of human fibroblast cells in 2D and 3D collagen gel cultures across a broad range of frequencies (10 Hz to 5 MHz) using a commercial well with in-plane electrodes. At low frequencies in both 2D and 3D models it was observed that protein adsorption influences the magnitude of the impedance for the cell-free samples. This effect was eliminated once cells were introduced to the systems. Cell proliferation could be monitored in 2D at intermediate frequencies (30 kHz). However, the in-plane electrodes were unable to detect any changes in the impedance at any frequency when the cells were cultured in the 3D collagen gel. The results suggest that in designing impedance measurement devices, both the nature and distribution of the cells within the 3D culture as well as the architecture of the electrodes are key variables.

## 1. Introduction

Standard cell culture methodology involves growing cells in a flask with a flat surface and letting them attach and proliferate on this area. While simple and reproducible, this technique does not represent the actual physiological condition of the cells in the human body. In recent years, 3D cell culture has been able to replicate many aspects of in vivo cell biology [1,2,3]. There are a range of approaches to 3D cell culture, each with their own specific properties. Cells can be maintained in suspension and driven to form a spheroid, or using tissue samples and stem cell biology they can be differentiated to form organoids that replicate the cell diversity found in organs like the kidney. Both of these systems have been shown to be highly effective for testing both basic biological function and drug interactions. For example, when drugs are tested in 3D, it has been shown that the expression levels of drug-metabolising enzymes resemble that of native tissue [2,3]. A third approach involves the use of a scaffold or matrix to support cell attachment and engineer the tissue architecture. The choice of matrix in which the cells grow is critical in determining cell morphology and function [2,4]. These systems can be designed to replicate the spatial organisation, cell population, cell diversity and function of human tissues in a way that cannot be readily achieved in 2D monolayer cultures and create larger tissue samples than can be achieved readily with organoids and spheroid cultures.

However, as 3D cell culture makes its way into laboratories, the majority of monitoring tools are still designed for 2D culture systems. Bright-field microscopy, a workhorse for cell monitoring in 2D, is not suitable for 3D cultures where the cells are usually embedded in a less-transparent extracellular matrix such as collagen and hydrogel matrices. End-point histology and fluorescence microscopy are currently the most commonly used tools in 3D cell culture [5]. Even though these techniques are gold standards, they are time-consuming and, in most cases, require the destruction of the sample for sectioning and staining. While live cell approaches abound, reporter constructs and stains have a limited lifespan and the architecture and scattering associated with 3D cultures makes the adaptation of traditional detection techniques challenging [6].

An alternative in situ approach for monitoring cell cultures involves the characterisation of electrical properties of cells in culture. Electrical impedance spectroscopy (EIS) is a well-established technique that is widely used to monitor cell proliferation, migration, and viability in 2D cultures [7,8,9,10]. This is a low-cost, label-free and fast-readout technique where electrodes are usually positioned beneath the culture and changes in current/voltage through the cells are measured as a frequency sweep is undertaken. This process allows the electrical resistance and capacitance of the cells to be monitored across the frequency spectrum [11,12]. Moreover, the whole spectrum can be studied and correlated with different parts of the cell culture system. Researchers have started applying this technique with 3D cultures [12,13,14,15,16,17,18,19]. Lei et al. and Pan et al. successfully detected cell proliferation and death of cells within 3D spheroids [15,16,17,18]. Further, Lee et al. were able to monitor single cells embedded in a hydrogel using vertical parallel electrodes positioned on either side of the culture [14]. Nonetheless, the application of EIS in 3D systems is complex. These models have several components that are continuously changing over the culture time (cells, matrix and medium), and one should be careful when interpreting EIS data as there is not yet a clear understanding of the effects of these changes on the impedance. Detailed reviews of the challenges involved in implementing EIS in 3D cultures have been recently written by our group and Gerasimenko et al. [20,21].

The cell index (CI) has been introduced by scientists to normalise the impedance values for data analysis. This allows for better analysis of the cell data as the background from control measurements is subtracted [18,20]. Further, it gives biologists an easier readout from their 2D systems that they can then correlate with cell proliferation and death. Even though application of the CI seems to be straight forward in 2D cultures, there are still some drawbacks in the use of the CI to analyse impedance based sensing. When CI is used to normalised the results the most sensitive frequency is singled out and the whole spectrum is discriminated as a result, this can lead to biases when choosing the most sensitive frequency. Further, in 3D cultures the lack of understanding of the individual components in the impedance spectrum means use of the CI to analyse 3D measurements is not straight forward and could lead to over-interpretation of results. The CI relies on the use of reproducible control samples and in 3D this become more challenging due to the nature of the matrix, cell distribution and the growth behaviour of the specific cell type. If the matrix in the absence of cells is used as the control one risks misinterpreting results if the matrix is not stable over-time or if its fundamental properties change in the presence of cells (e.g., contraction, deposition or turnover of extracellular matrix and/or matrix degradation).

The goal of this study was to understand how the distribution and architecture of cells in 2D and 3D affects impedance measurements gathered with an in-plane electrode. The 3D culture used in this study was a dermis model with fibroblast cells embedded in a collagen matrix [22]. We analysed and compared the changes observed in both 2D and 3D cultures and studied their relationship to the cells and components present in the system. X-ray photoelectron spectroscopy (XPS) was used to examine the surfaces of the electrodes and bright field or fluorescence microscopy to examine the cells’ location during culture, allowing these parameters to be correlated with changes in the impedance spectrum and the associated CI.

## 2. Materials and Methods

### 2.1. Cell Culture

Human telomerase reverse transcriptase (hTERT) immortalised foreskin human fibroblast cells (BJ-5Ta, CRL-4001) were purchased from ATCC (American Type Culture Collection) (Manassas, VA, USA). The cells were cultivated in 4:1 mixture of Gibco Dulbecco’s modified Eagle’s medium (DMEM) and Gibco Medium 199 supplemented with 10% fetal bovine serum (FBS) (Sigma-Aldrich, Darmstadt, Germany) and 0.01mg/mL hygromycin B under standard conditions (37 ${}^{\circ }$C, 5% CO${}_{2}$). Sub-cultivation was done in a 1:10 ratio. Cells were detached from culture flasks by treatment with trypsin-EDTA (0.25%, Life Technologies, Darmstadt, Germany) for 5 min. After detachment, cells were re-suspended in their cultivation medium to stop any remaining trypsin activity. After centrifugation at 250× *g* for 5 min, the supernatant was removed and the cells were re-suspended in the cultivation medium. Cells were used in passage 5 to 20.

### 2.2. Impedance Measurement System

A commercially available electric cell-substrate impedance sensing (ECIS) electrode array with integrated wells was purchased (ECIS-8W1E PET, Applied BioPhysics, NY, USA). The system consists of an array of gold electrodes deposited on a polyethylene terephthalate (PET) sheet, which is glued to 8 wells with a surface area of 0.8 cm${}^{2}$. The working electrode has a diameter of 250 μm. The wells were supplied sterile by the manufacturer. Before every experiment, each electrode was treated by adding 200 μL of 10 mM L-cysteine (Sigma-Aldrich) in reverse osmosis (RO) water for 10 min at room temperature, then rinsed twice with RO water as recommended by the manufacturer.

The impedance spectrum was recorded every 24 h. To stabilise the temperature of the medium and cells, before every measurement the well was left at room temperature for 30 min. The impedance spectrum was collected from 10 Hz to 5 MHz. To obtain the impedance of the system, a small sinusoidal voltage (10 mV) was applied for a few milliseconds. Electrical impedance spectroscopy measurements were performed using a MFIA Impedance Analyser (Zurich Instruments, Zurich, Switzerland). The instrument was controlled using custom software initially developed by Dr. Steve Beguin and later adapted to this work in MATLAB R2016b (MathWorks, USA). The data was analysed using Python 3 in a Jupyter notebook. Here, the cell index (CI) is defined as

(1) $$\mathrm{CI}=\frac{|Z_{cell}\left(t\right)|-|Z_{control}\left(t\right)|}{|Z_{control}\left(t\right)|},$$

where $|Z_{cell}\left(t\right)|$ is the impedance magnitude of the samples containing cells at time *t* and $|Z_{control}\left(t\right)|$ is the impedance magnitude of the samples used as a control at time *t*. The controls were DMEM + FBS for the measurements in 2D and collagen in DMEM + FBS for the measurements in 3D.

### 2.3. Cell Culture on Electrodes

For the 2D measurements, $3\times 10^{4}$ fibroblast cells were suspended in 500 μL of their culture medium and seeded on top of the electrodes. Cells were allowed to attach to the electrode surface and then measurements were collected every 24 h over a total of 7 to 10 days.

The collagen matrix was prepared so that the final concentration of collagen was 3 mg/mL. The application note for KerCT Immortalized Keratinocyte Cells (CRL–4048^TM^, ATCC, Manassas, VA, USA) was used to prepare the collagen matrix. Stock reagents and final concentrations are shown in Table 1.

For the 3D dermis model, 200 μL of the collagen matrix solution was prepared as described above with $3\times 10^{4}$ fibroblast cells mixed into the collagen prior to gelling for 30 min in standard cell culture conditions (37 ${}^{\circ }$C, 5% CO${}_{2}$) and then topped up with cell culture medium. For each experiment two or three wells were seeded with cells in collagen and topped up with DMEM, while two or three wells were seeded with cell-free collagen and topped up with DMEM to serve as controls. The impedance spectrum was then collected every 24 h for 10 days in a frequency range from 50 Hz to 5 MHz for the three biological replicates.

### 2.4. Cell Imaging

Bright field images were taken using either ZEISS Axio Vert.A1 and ZEN 2010 (Carl Zeiss Microscopy GmbH, 2011) or Nikon Eclipse T*i* with NIS-Elements Advanced solutions (Nikon). For the phalloidin staining the 3D samples were first fixed in 3.7% formaldehyde for 30 min on a shaker, followed by washing three times in 1× PBS, each time for 5 min on a shaker. They were then permeabilised using 0.1% Triton X-100 for 30 min at room temperature on a shaker. The tissues were washed three times for 5 min with PBS on a shaker and incubated with Alexa Fluor^TM^ 488 Phalloidin (Invitrogen, Carlsbad, CA, USA) for 30 min for F-actin staining. Confocal images of 3D tissues were taken using Nikon A1 motorised inverted confocal microscope and NIS-Elements Advanced Research (Nikon, Tokio, Japan) with an emission wavelength of 525 nm and excitation of 488 nm.

### 2.5. X-ray Photoelectron Spectroscopy (XPS) Measurements

X-ray photoelectron spectroscopy (XPS) analysis was carried out on the commercial (ECIS) wells. Two working electrodes were left intact supplied by the manufacturer. Six wells were treated with L-Cysteine with two then incubated in Dubelcco’s phosphate-buffered saline (DPBS), two with cell medium and two with a collagen gel topped up with cell medium. All the electrodes were incubated for 7 days under standard cell culture conditions (37 ${}^{\circ }$C, 5% CO${}_{2}$). After 7 days the collagen and medium were removed and the wells were washed with reverse osmosis (RO) water three times, the wells were then dried under a stream of nitrogen before XPS measurements were taken.

X-ray photoelectron spectroscopy (XPS) analysis was performed using an AXIS Nova spectrometer (Kratos Analytical Inc., Manchester, UK) with a monochromated Al Kα source at a power of 180 W (15 kV × 12 mA) and a hemispherical analyser operating in the fixed analyser transmission mode. The total pressure in the main vacuum chamber during analysis was typically between 10${}^{-9}$ and 10${}^{-8}$ mbar. Survey spectra were acquired at a pass energy of 160 eV and step size 0.5 eV. To obtain more detailed information about chemical structure, oxidation states etc., high resolution spectra were recorded from individual peaks at 40 eV pass energy and a step size of 0.1 eV, typically yielding a FWHM of <0.8 eV for the Ag 3d${}_{5/2}$ peak and <0.85 eV for the carbon 1s ester peak of polyethylene terephthalate (PET) during performance tests.

The samples were analysed at a nominal photoelectron emission angle of 0${}^{\circ }$ w.r.t. the surface normal. Since the actual emission angle is ill-defined in the case of rough surfaces and powders as in the present case (ranging from 0${}^{\circ }$ to 90${}^{\circ }$) the sampling depth may range from 0 nm to approx. 10 nm. In a first step the standard aperture slot was used for analysis in which case the area analysed on the sample is elliptical in shape with approximate dimensions of 0.3 mm × 0.7 mm. In a second step (repeat analysis) the sampling area was reduced to a 220 μm spot using an aperture.

Data processing was performed using CasaXPS processing software version 2.3.21 (Casa Software Ltd., Teignmouth, UK). All elements present were identified from survey spectra. The atomic concentrations of the detected elements were calculated using integral peak intensities and the sensitivity factors supplied by the manufacturer. Binding energies were referenced to the C 1s peak at 285.0 eV (“neutral” hydrocarbon). The accuracy associated with quantitative XPS is ca. 10–15%. Precision (ie. reproducibility) depends on the signal/noise ratio but is usually much better than 5%. The latter is relevant when comparing similar samples.

## 3. Results

### 3.1. 2D Cultures

**Figure 1:**
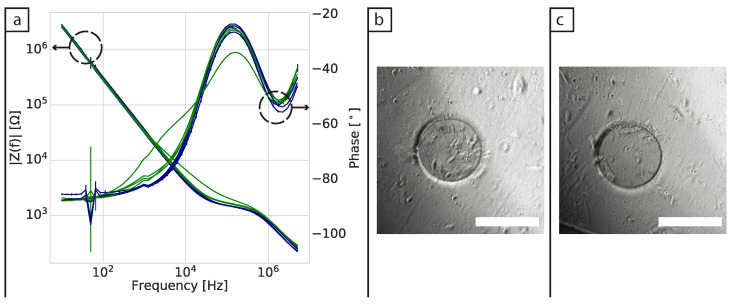
(**a**) Impedance spectra of BJ-5Ta cells 24 h after seeding in 2D. Blue lines are from control wells containing Dulbecco’s modified Eagle’s medium (DMEM) + fetal bovine serum (FBS) cell medium, while green lines are from wells containing cells and cell medium. (**b**,**c**) Bright field images of two of the wells seeded with cells after 24 h of culture. Scale bars = 250 μm.

Impedance spectra for fibroblast cells seeded directly on the surface of the electrode (2D model) were collected every 24 h for either 7 or 10 days in a frequency range from 10 Hz to 5 MHz for two biological replicates and a total of 4 samples. Equivalent wells were run without cells as controls. Figure 1a shows the impedance phase and magnitude after 24 h of culture. It was observed that in 3/4 of the samples there was no clear change in the signal from the wells containing cells compared to the control in either the magnitude or the phase spectra. In one well containing cells the magnitude was observed to increase between 10 kHz and 1 MHz, which is the frequency range typically associated with cell attachment in 2D impedance measurements [11].

The phase spectra showed that there is a shift in the phase from the same well (Figure 1a). Here two changes from capacitive (−90${}^{\circ }$) to more resistive (approaching 0${}^{\circ }$) behaviour were observed: one before 100 kHz and one after 1 MHz. In previous work, these changes have been related to the coating on the surface of the electrodes combined with the double layer capacitance and the solution resistance, together with the spreading resistance of the exposed gold region [23]. A noise peak at 50 Hz is visible in both the magnitude and phase in all of the impedance measurements, due to interference from the mains supply. Bright field images from the surface of the wells (Figure 1b,c) showed that after 24 h the distribution and number of cells across the working electrode varied. Figure 1b corresponds to the electrode where changes in both impedance magnitude and phase were detected, illustrating how these early attachment processes influence the measurements. Microscope pictures of the other biological replicates are shown in Figure S1.

**Figure 2:**
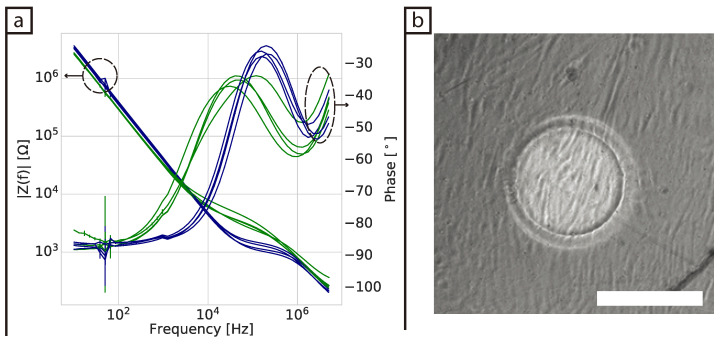
(**a**) Impedance spectra of BJ-5Ta cells seven days after seeding in 2D. Blue lines are from control wells containing DMEM + FBS cell medium, while green lines are from wells containing cells and cell medium. (**b**) Bright field image of a well seeded with cells after seven days of culture. Scale bar = 250 μm.

Figure 2a shows the spectrum 7 days after seeding. The overall impedance magnitude of the wells containing cells has visibly increased in the range from about 10 kHz to 1 MHz and they have all reached similar magnitude values across the spectrum. The variability in the phase between replicates had increased, this spreading is consistent through the frequencies with the major changes starting at 10 kHz. There is also a major frequency shift in the first dispersion of the system from approximately 160 to 40 kHz. Figure 2b shows a typical bright field image of confluent cells covering the surface of an electrode after 7 days of culture (see Figure S2 for images of the other replicates).

**Figure 3:**
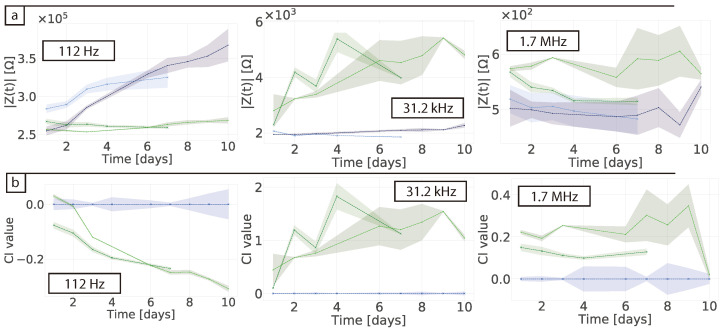
(**a**) Impedance magnitude and (**b**) cell index (CI) changes over-time at 112 Hz, 31.2 kHz and 1.74 MHz. Measurements were taken daily while the electrodes were incubated in standard culture conditions. For the CI the impedance was normalised to the average of the samples containing DMEM + FBS. Blue lines are from wells containing DMEM + FBS medium and green lines are from wells seeded with $3\times 10^{4}$ cells in medium. Shaded area shows minimum and maximum of the replicates.

The impedance magnitude was plotted against time at specific key frequencies to investigate how the sensor response varied over the culture period. As shown in Figure 3a, at 112 Hz there was a steady increase in the impedance magnitude over the course of 10 days for the cell-free wells, while the wells with cells remained relatively stable. In contrast, at 31.2 kHz the impedance of the cell-free samples remained stable, but the impedance tended to increase over time when cells were present. At 1.7 MHz there were very few changes in the data, which is expected as these high frequencies measure the bulk electrolyte and would not be expected to exhibit major changes over time [11].

Figure 3b shows the cell index (CI) data, which is normalised to the average of the cell-free control wells according to Equation (1). At 112 Hz, sample-to-sample variations in the normalised cell-free samples became apparent, making it difficult to draw conclusions or interpret the data in great detail. Nevertheless, a clear negative trend was observed in the wells containing cells. On average at 31.2 kHz the CI was stable in the absence of cells and where cells were present the CI rose as the cells proliferated and then plateaued as they reached confluence, but varied over time and between samples. This is shown by the shaded area in the figure where there is variability between replicates that relates to the difference in cell growth and mobility between the different wells as correlated with microscopy images. Previous work has demonstrated that CI generally correlates linearly with cell number on the surface of the electrode [10,24,25].

### 3.2. Three-Dimensional Cultures

**Figure 4:**
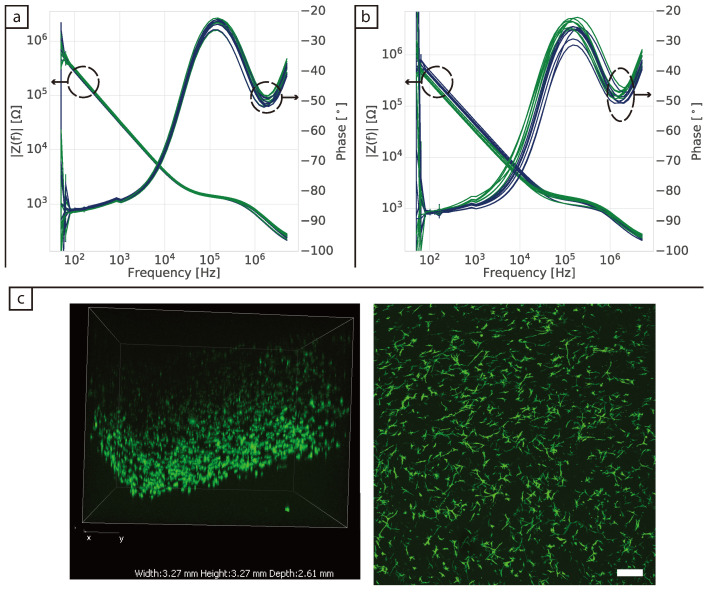
Impedance spectra of BJ-5Ta cells (**a**) 24 h and (**b**) eight days after seeding in 3D. Dark blue lines are from control wells containing collagen with DMEM + FBS medium, while dark green lines are from wells containing cells in collagen and cell medium. (**c**) Confocal pictures of collagen seeded with 3 × 10${}^{4}$ cells fixed after 24 h of culture and stained with phalloidin. (**left**) Side view of 3D confocal volume and (**right**) maximum intensity projection in the XY plane. Scale bar represents 250 μm.

After key frequencies for cell detection in 2D were identified, the 3D dermis model was introduced to the wells. Figure 4a,b show the impedance spectra of the cells in collagen after 24 h and 8 days of culture, respectively. No major changes in the impedance of cells were observed in either of the impedance components after 24 h, with confocal pictures (Figure 4c) showing that the cells were distributed throughout the collagen and did not contact each other. After 8 days of culture, sample-to-sample variability in the phase spectra became more apparent as shown in Figure 4b, irrespective of whether there were cells present in the collagen or not. Increased sample-to-sample variation was also observed in the magnitude signals after 8 days. These changes occurred across the frequency range with no clear trends emerging between the samples. Noise due to the mains supply was again visible at 50 Hz.

**Figure 5:**
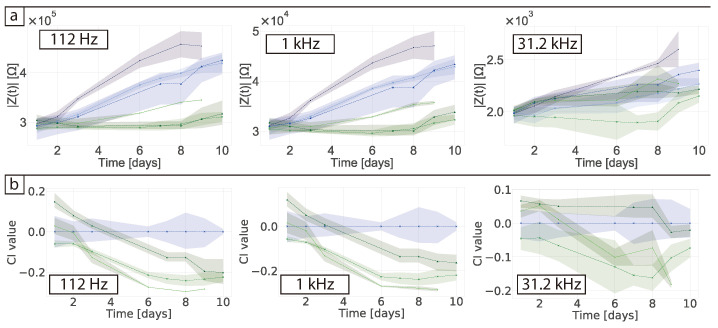
(**a**) Impedance magnitude and (**b**) cell index (CI) changes over time for measurements taken daily at 112 Hz, 1 kHz and 31.2 kHz while the electrodes were incubated in standard culture conditions. The impedance was normalised to the average of the samples containing collagen and DMEM + FBS. Blue lines are from wells containing collagen in DMEM + FBS medium and green lines are from wells seeded with $3\times 10^{4}$ cells in collagen and medium. Shaded area shows minimum and maximum of the two replicates.

When the impedance magnitude was plotted against time at selected frequencies (Figure 5a), a steady increase in impedance magnitude was observed at low frequencies for the cell-free collagen samples. At 112 Hz, one of the biological replicates containing cells increased at a slower rate, while for the other two the magnitude remained stable over-time. Similar to the results in 2D, the cells appeared to stabilise the impedance magnitude up to 1 kHz. To check if the cell number influenced the outcome, seeding densities of $3\times 10^{4}$ and $15\times 10^{5}$ cells were compared, but the impedance magnitude remained equally stable over-time at low frequencies (see Figure S3). At 30 kHz, there was little change in the impedance magnitude over-time (note differing scales on the y-axes in Figure 5a). From the 2D experiments it was determined that 30 kHz was the most sensitive frequency for the cells, whereas in 3D no specific trend related to cell proliferation was observed at any frequency. Figure 5b shows the normalised impedance magnitude over-time. At low frequencies (112 Hz and 1 kHz) the CI decreased by a maximum of ≈0.3 Ω, which is similar to the changes observed in 2D for these cells. This indicates that these changes were unaffected by the culture methods and the changes in cell morphology. At higher frequencies no clear trends were observed regardless of the amount of cells seeded.

**Figure 6:**
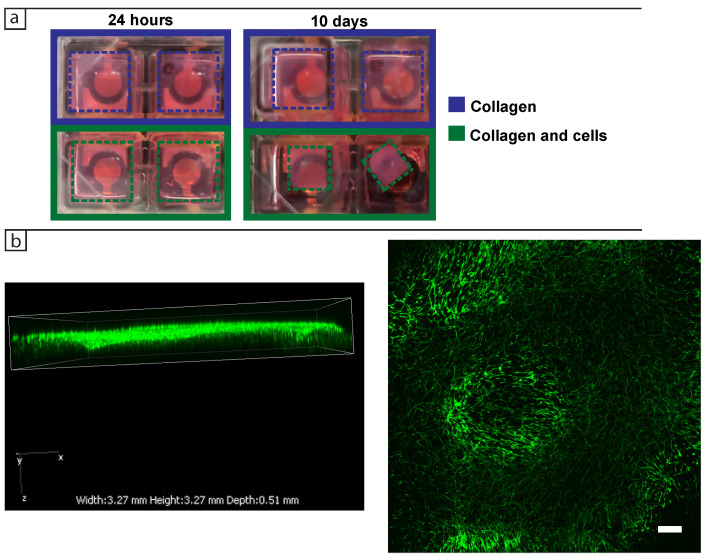
(**a**) Pictures of the wells seeded with $3\times 10^{4}$ cells in collagen on the electrodes after 24 h (**left**) and 10 days (**right**). Blue dashed lines show the control samples containing only collagen in DMEM + FBS, while the green dashed lines show the samples containing cells in collagen and DMEM + FBS. (**b**) Confocal pictures of collagen seeded with 3 × 10${}^{4}$ cells fixed after 10 days of culture and stained with phalloidin. (**left**) Side view of 3D confocal volume and (**right**) maximum intensity projection in the XY plane. Scale bar represents 250 μm.

In the 3D model the cells contracted the collagen as they proliferated. As shown in Figure 6a, the tissue had contracted to ≈50% of its original size after 10 days. This contraction may contribute to the variability of the impedance signal as it seems to have a stochastic behaviour [26,27] and no straightforward means of controlling it. There are more cells visible in the confocal images after 10 days of culture (Figure 6b), but it is not clear whether this is because there are more cells in the tissue or the same number of cells are occupying a smaller volume. Note that the contraction is visible in the Z axis as well. This variability presents a challenge for the impedance measurements and their subsequent validation.

Due to the contraction of the tissues, they tended to float in the well. The resulting changes in position of the collagen are likely to contribute to the overall variability and lack of sensitivity of the impedance measurements in this 3D model.

### 3.3. Understanding Impedance Magnitude Changes at Low Frequencies

To understand the impedance changes detected at low frequency in the cell-free 2D and 3D samples, a series of experiments were run to monitor the system over 10 days of cell-free operation. Electrodes were examined with increasingly complex media starting with Dubelcco’s phosphate-buffered saline (DPBS), then Dulbecco’s modified Eagle’s medium (DMEM) and finally DMEM + serum (FBS). Matching wells were also tested with the 3 mg/mL collagen gels in the same solutions. The impedance spectra were collected every 24 h for 10 days in a frequency range from 10 Hz to 5 MHz with two replicates.

**Figure 7:**
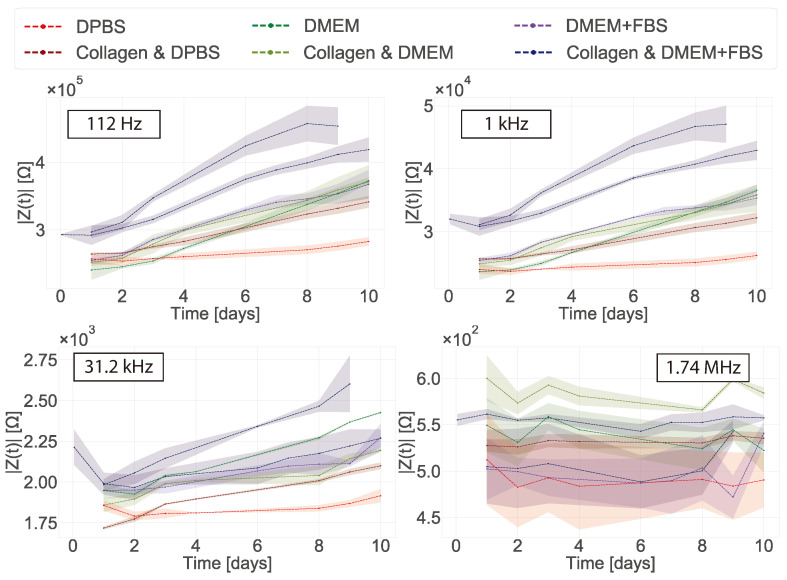
Impedance magnitude plots showing measurements taken daily at 112 Hz, 1 kHz, 31.2 kHz and 1.74 MHz while the electrodes were incubated in standard culture conditions (37 ${}^{\circ }$C, 5% CO${}_{2}$) with and without collagen gels in Dubelcco’s phosphate-buffered saline (DPBS), DMEM, DMEM + FBS. The shaded areas show minimum and maximum of the two replicates.

The impedance magnitude was plotted over time for discrete frequencies (Figure 7). At low frequency (112 Hz) the impedance magnitude increased over-time for all of the samples, with DPBS displaying the most stable value. As low frequencies are directly related to the impedance of the double layer capacitance of the electrode, these observations may arise from changes occurring at the surface of the electrodes [28]. Therefore we hypothesised that this increase in magnitude over time was due to the deposition of materials from the medium (proteins, macromolecules) onto the surface of the electrode. These effects were still observed at 1 kHz and 31.2 kHz, with DPBS and collagen in DPBS having a lower impedance than the rest of the samples. However, the effect becomes less pronounced at higher frequencies (note the different y-axis scales in Figure 7). At higher frequencies (1.74 MHz) no clear trend identified, which is expected as the bulk electrolyte has similar electrical conductivity in each case.

**Figure 8:**
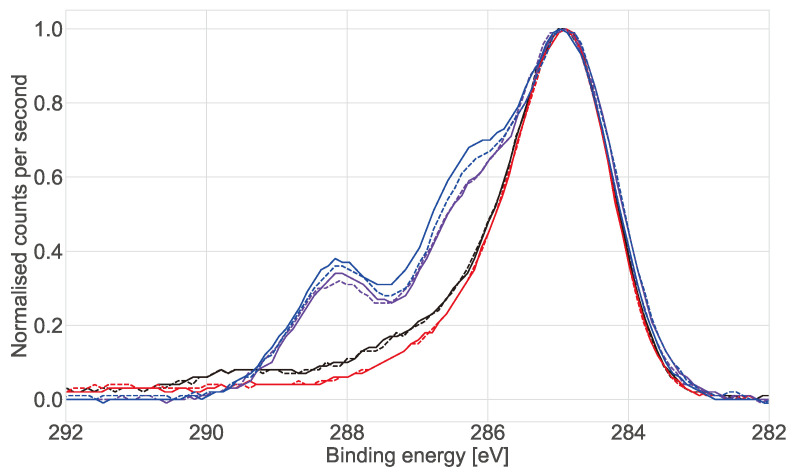
Normalised C 1s XPS spectra of the working electrode after 7 days of incubation in standard culture conditions (37 ${}^{\circ }$C, 5% CO${}_{2}$). Black lines show the bare gold electrode exposed to air. Red lines show the electrode exposed to DPBS, purple lines show exposure to DMEM + FBS and blue lines show collagen gel in DMEM + FBS, these electrodes were cleaned with L-cysteine prior to exposure to the aqueous media.

X-ray photoelectron spectroscopy (XPS) was performed on the working electrodes after they were exposed to the different media and buffer solutions to investigate the composition of the electrode surfaces in more detail. Analysis of the elemental components (see Table S1) showed that the wells had silicone contamination that was cleaned off with the L-cysteine treatment and that the nitrogen content of the surface rose to 12% after exposure to the medium and FBS. Figure 8 shows the XPS spectra of C 1s on the different electrodes after 7 days of incubation in standard culture conditions. The C 1s spectra for the electrodes exposed to DMEM show two additional peaks at ≈288.1 eV and ≈286.1 eV, which alongside the increase in the nitrogen content of the surface suggests the deposition of amine- and amide-rich materials (proteins) [29].

## 4. Discussion

Impedance spectroscopy has been widely characterised and studied for its use in cell culture since the 90s [7,8]. Research using small working electrodes and interdigitated electrodes has shown that it is possible to monitor cells in two dimensional (2D) cultures using this technique. While small working electrodes have been used to monitor confluent cell cultures challenged with drugs, interdigitated working and reference electrodes have been shown to be more effective at monitoring cell proliferation across the well surface over time [7,10,25,30]. This highlights the importance of electrode configuration for monitoring cell cultures and the differences in their potential applications. We have studied the use of a commercially available well with a small working electrode to monitor fibroblast cells in 2D and 3D. We have also measured the impedance across a broad frequency range (10 Hz to 5 MHz), rather than just the typically sensitive frequency of ≈10 kHz used in 2D cultures.

In 2D cultures the impedance magnitude of the control samples (DMEM + FBS only) increased over time in the low frequency range (10 Hz to 1 kHz), reflecting an increase in protein adsorption at the surface of the electrodes. In contrast, a stable signal was observed for the samples with cells. This decrease of impedance magnitude of cell samples relative to cell-free controls at low frequencies has been observed in 2D before [31]. However, no clear explanation was given to this phenomenon. Here we proposed that protein adsorption from the medium was the cause and confirmed it with XPS measurements. This effect is most visible in the lower frequency range (<10 kHz). We hypothesise that the presence of the cells in the culture serves to stabilise the surface of the electrode, hence controlling protein adsorption (though not eliminating it). At mid-frequencies (1 kHz to 100 kHz) an increase related to the presence and proliferation of the cells was observed. However, there was variation between replicates and the increases reflected the number of cells on the surface of the individual working electrode. This highlights why confluent layers are more commonly used as controls when using impedance in 2D as they ensure coverage of the whole electrode and well area [10,30]. Furthermore, validation of impedance measurements in 2D is relatively straightforward, as the surface of the electrodes can usually be viewed directly with a brightfield microscope. This adds to the challenges of translating the technique to 3D cultures, where validation is done mainly via the analysis of replicates, rather than measuring the same cultures post impedance measurement due to the need to fix and/or stain samples prior to imaging.

Some challenges were encountered when the in-plane ECIS device was used to monitor fibroblast cells in a collagen gel (3D model). First, the collagen contracts as the cells proliferate in the gel and this appears to generate additional variability in the measurements. However, at low frequencies protein adsorption was still observed for the control samples (DMEM and collagen gel), but not for the samples containing cells. It is not clear why no protein adsorption is observed in the samples containing cells as no cells were present at the surface of the electrodes. To the best of our knowledge there have been no impedance studies of fibroblast cells immerse in a collagen gel before. Pan et al. [17,18] and Lei et al. [16] were able to monitor cell proliferation of 3D spheroids using parallel and interdigitated electrodes, respectively. However, their 3D cell culture model was fundamentally different as it consisted of multiple spheroids with each spheroid consisting of a cluster of cells connected via tight junctions. Thus, one could expect this system to have a higher resistance compared to our system where we are attempting to monitor dispersed single cells spread throughout a gel.

In their study Pan et al. used an absolute value of the CI to determine the difference between their controls and the samples containing cells, ascribing growth with CI value differences no larger than 0.6 [17,18]. Here we obtained a CI of 0.3 that can be attributed to protein adsorption. When the cells were measured in 2D a clear CI of ≈2 was obtained in our studies and ≈1.5 in Pan et al. This shows that overall the sensing system is not as sensitive in 3D as they are in 2D. Furthermore, the authors found that the proliferation of the cells decreased the impedance magnitude, which is the same effect we saw in our normalised data and showed the effect of protein adsorption. Nonetheless, the authors measured the stability of their matrigel alone for a period of 3 days and the CI variability was of around 0.02. The authors also found a sensitive frequency of 25 kHz which is not related to protein adsorption. This suggests that the small differences they observed were indeed from the cells. When our data was normalised to obtain the CI, the effects of protein adsorption in the cell-free controls were lost and results could be misinterpreted as measurements of the cells themselves. One should be careful when normalising data so no valuable information from the system is lost and data is not over-interpreted. In this regard, choosing and monitoring the temporal changes of the right controls is crucial when performing impedance measurements. Tonello et al. used a different equation to calculate the CI, namely $CI_{\left(n\right)}=|I_{cells(n)}\left|-\right|I_{cells(n-1)}\left|-\right|I_{nocells(n)}\left|-\right|I_{nocells(n-1)}|$ [32]. In this case they were looking at time point variations by subtracting the impedance of the cells at the different time points and comparing them to the gel in the absence of cells. They found their CI increased over-time with as the cells proliferated. When our data was normalised using this equation no clear trend was observed and a negative CI value was obtained (see Figure S4).

**Figure 9:**
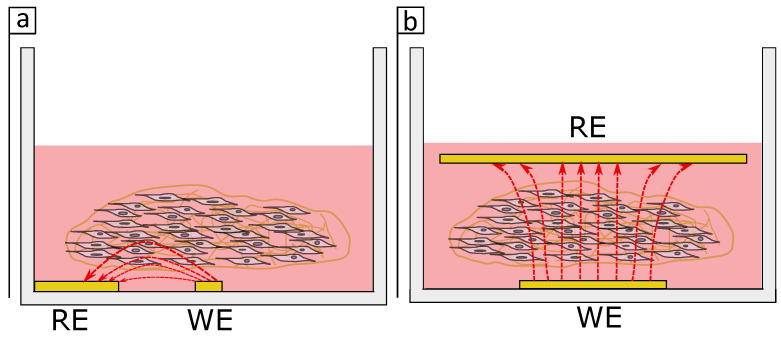
Schematic drawing showing the current flow (red arrows) between working electrode (WE) and reference electrode (RE) when the collagen gel is in an (**a**) in-plane or in a (**b**) parallel electrode setup.

Lee et al. have used parallel electrodes to monitor single human breast cancer cells (GFP-MCF-7) encapsulated in alginate hydrogel [14]. However, they did not use the full impedance components of the spectrum but only the capacitance, and monitored the cells over-time using 1 kHz as their sensitive frequency. This is interesting because we were still observing the effects of protein adsorption in our system at 1 kHz, which makes it unclear if the changes observed in the earlier study were due to cell proliferation itself or protein adsorption. Note that it is also not clear whether Lee et al. immersed the cell-free hydrogels in cell medium or protein-free PBS, in which case the changes could be more easily correlated to cell proliferation. More recently, Tonello et al. measured the impedance of human mesenchymal stromal cells in gelatin-chitosan hybrid hydrogel scaffolds [32]. These cells did not form spheroids but rather individually proliferated within the hydrogel. Similar to our results, the authors found that the impedance of the cell-free hydrogels was changing over time and this phenomenon was different when the cells were present in the system. They attributed this behaviour to the medium hydrating the hydrogel scaffold and increasing the conductivity, hence decreasing the impedance of the system over time. This effect is opposite to the results observed in the present study, where we saw the impedance of the gel increasing over time, whereas in our case this effect was not related to the gel itself but to the medium. Moreover, Tonello et al. used 4 kHz and were able to monitor the cells at this frequency, whereas that was not the case in our study. The key difference between their setup and the one in this study is that they used parallel electrodes placed on each side of the gel, which may improve the sensitivity. Figure 9 shows a schematic drawing of the current flow in an in-plane and parallel electrodes setup. While for the in-plane setup (Figure 9a) the current may penetrate only a small volume of the tissue when the electrodes are placed in parallel (Figure 9b) a higher volume of the tissue is penetrated by the current which in hand may have a bigger impact in the impedance measurements. It appears the electrodes dimensions and design are critical for monitoring distributed cells in a 3D tissue.

Overall, this study shows that commercially available in-plane electrodes are not suitable for monitoring 3D cultures. The complexity of the cell distribution in addition to the penetration of the electrical signals into the 3D culture represents a challenge for the direct translation of conventional impedance systems to work with 3D cultures. While researchers have been able to monitor 3D spheroids, the CI differences are still small and could be misleading if normalisation is done without caution. However, in our system it was not possible to monitor single fibroblast cells embedded in a collagen gel using electrodes on the surface of the culture well. There are too many components changing in this 3D systems (matrix, cells and medium) which makes it even more difficult to understand what is happening in the system. Different electrodes architectures have been shown to be significant in the monitoring of cells in 3D cultures, and may aid with the monitoring of the 3D model presented in this work. Furthermore, the contraction of the tissue represents a challenge for the reproducibility of both the 3D model itself and its consequent impedance measurement. Future work on modelling different electrode configurations should provide insight into how changes in electrode design will influence the measurement of 3D cultures and determine how electrodes can be designed to ensure current penetrates through the tissue and in turn improve the sensitivity.

## 5. Conclusions

We have investigated the potential use of established 2D culture EIS methods in 3D cell cultures. We found that the setup even though ideal for 2D cultures is not adequate for monitoring cell proliferation in our 3D collagen gel model. When attempting to monitor 3D cell cultures with EIS one should be careful to use the right controls and confirm their stability over-time, in order to not over-interpret results or misinterpret CI values. It is apparent from this data that in plane electrodes lack the ability to penetrate across the cell culture volume, a factor that may be able to be addressed by redesigning the system with parallel electrodes or other distributed electrode arrays.

## Figures and Tables

**Table 1:** Reagents and concentrations to prepare collagen matrix.

Reagent	Stock Concentration	Final Concentration	Volume [μL]
Eagle’s Minimum Essential Medium (EMEM, Gibco)	10×	0.84×	84
FBS	100%	9.28%	92.8
L-Glutamine (Gibco)	200 mM	1.48 mM	7.4
Sodium Bicarbonate (Gibco)	7.5%	0.28%	37.3
Collagen Type I (Corning)	9 mg/mL	3 mg/mL	333.3
BJ-5Ta culture medium			445.1
		**Final Total Volume**	**1 mL**

## Data Availability

The data presented in this study is available on request from the corresponding author.

## Supplementary Materials

The following are available at https://www.mdpi.com/2306-5354/8/1/11/s1, Figure S1. Bright field pictures of the wells seeded with cells after 24 h of culture, scale bars represent 250 µm. (a) and (b) are one biological replicate, while (c) and (d) are the other one. Red arrows point the cells on the surface of the electrode, Figure S2. Bright field pictures of the wells seeded with cells after 7 days of culture, scale bars represent 250 µm. (a) and (b) are one biological replicate, while (c) and (d) are the other one, Figure S3. Impedance magnitude plots depicting measurements taken daily at (a) 112 Hz, (b) 31.2 kHz and (c) 1.74 MHz while the electrodes were incubated in standard culture conditions. (a,b,c-i) show the impedance magnitude of the control replicates at the different frequencies, blue lines are from wells containing collagen in the media DMEM + FBS. (a,b,c-ii) show the averaged impedance magnitude of the control replicates in blue line at the different frequencies, green lines are from wells seeded with $3\times 10^{4}$ cells in collagen as well as the media. (a,b,c-iii) show the averaged impedance magnitude of the control replicates in blue line at the different frequencies, dark red lines are from wells initially seeded with $15\times 10^{5}$ cells in collagen as well as the media. The shaded area shows the minimum and maximum of the replicates in each experiment and the dashed lines show the slope fitted to each measurement, Table S1. Elemental data obtained from the X-ray photoelectron spectroscopy (XPS) measurements. Figure S4. Impedance magnitude plots depicting measurements taken daily at 112 Hz, 31.2 kHz and 1.74 MHz while the electrodes were incubated in standard culture conditions with 3D cultures. Data was normalised using $CI_{\left(n\right)}=|I_{cells(n)}\left|-\right|I_{cells(n-1)}\left|-\right|I_{nocells(n)}\left|-\right|I_{nocells(n-1)}|$. (**a**), (**b**), (**c**) are the different biological replicates.
