# Supplementary material for: Extending In-Plane Impedance Measurements from 2D to 3D Cultures: Design Considerations
Source: Bioengineering (Basel). 2021 Jan 13;8(1):11. doi: 10.3390/bioengineering8010011 (PMC7828367; doi:10.3390/bioengineering8010011)
Supplement: Supplementary file 1 [file bioengineering-08-00011-s001.pdf]

Supplementary Materials: Extending In-Plane Impedance Measurements From 2D To 3D Cultures: Design Considerations

Sorel E. De León [1,2], Lana Cleuren [3], Zay Yar Oo [1,2], Paul R. Stoddart [1] and Sally L. McArthur [1,2,*]

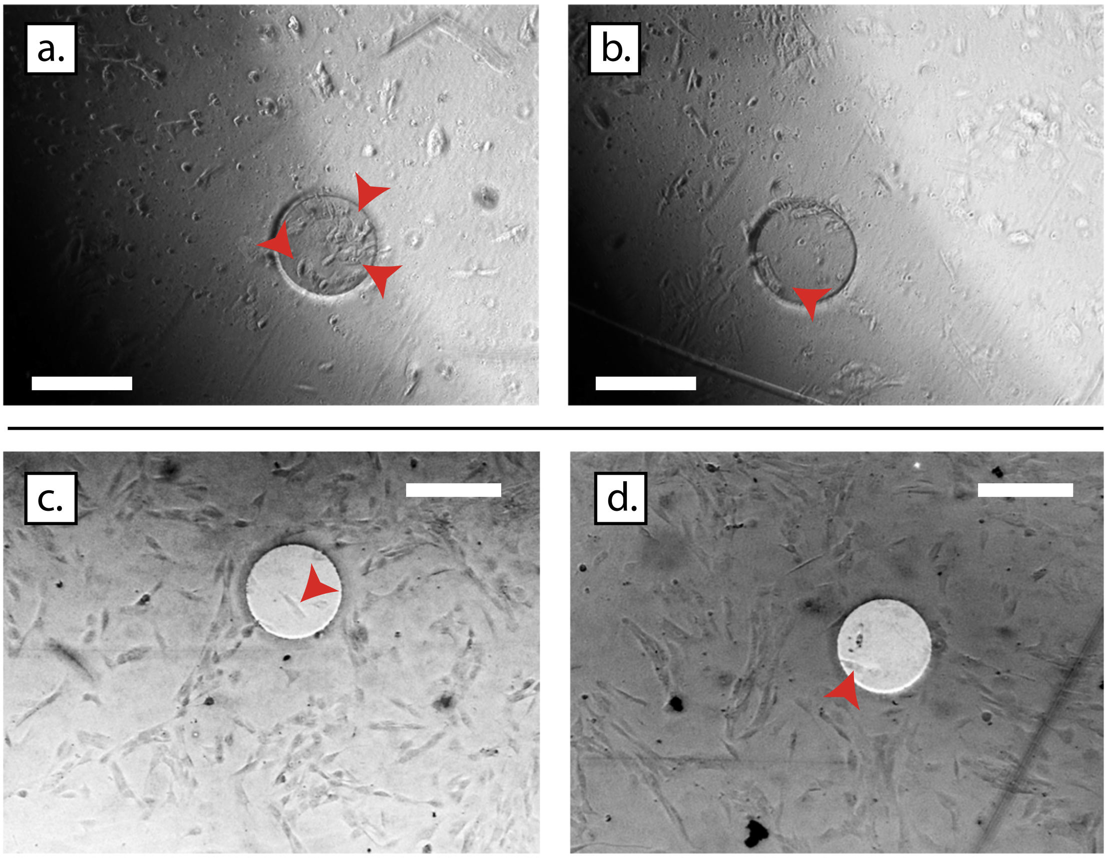

Figure S1. Bright field pictures of the wells seeded with cells after 24 hours of culture, scale bars represent 250 μm.(**a**) and (**b**) are one biological replicate, while (**c**) and (**d**) are the other one. Red arrows point the cells on the surface of the electrode.

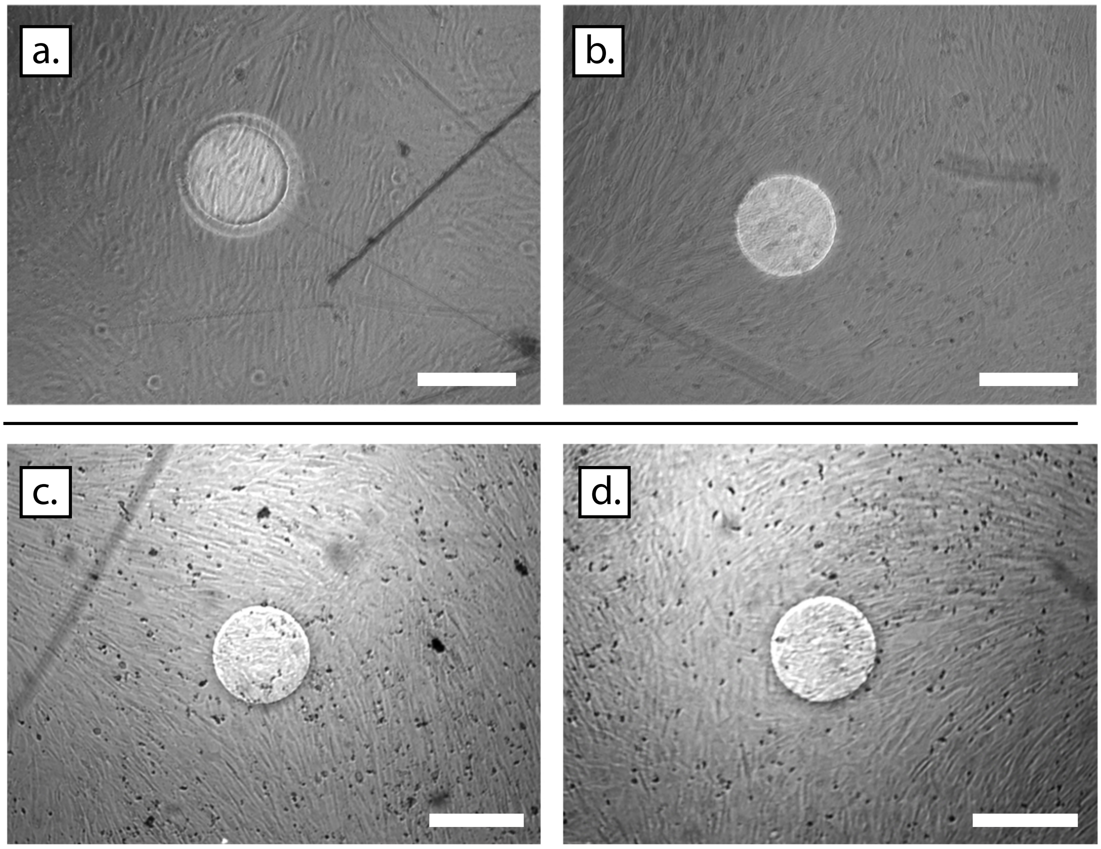

Figure S2. Bright field pictures of the wells seeded with cells after 7 days of culture, scale bars represent 250 μm.(**a**) and (**b**) are one biological replicate, while (**c**) and (**d**) are the other one.

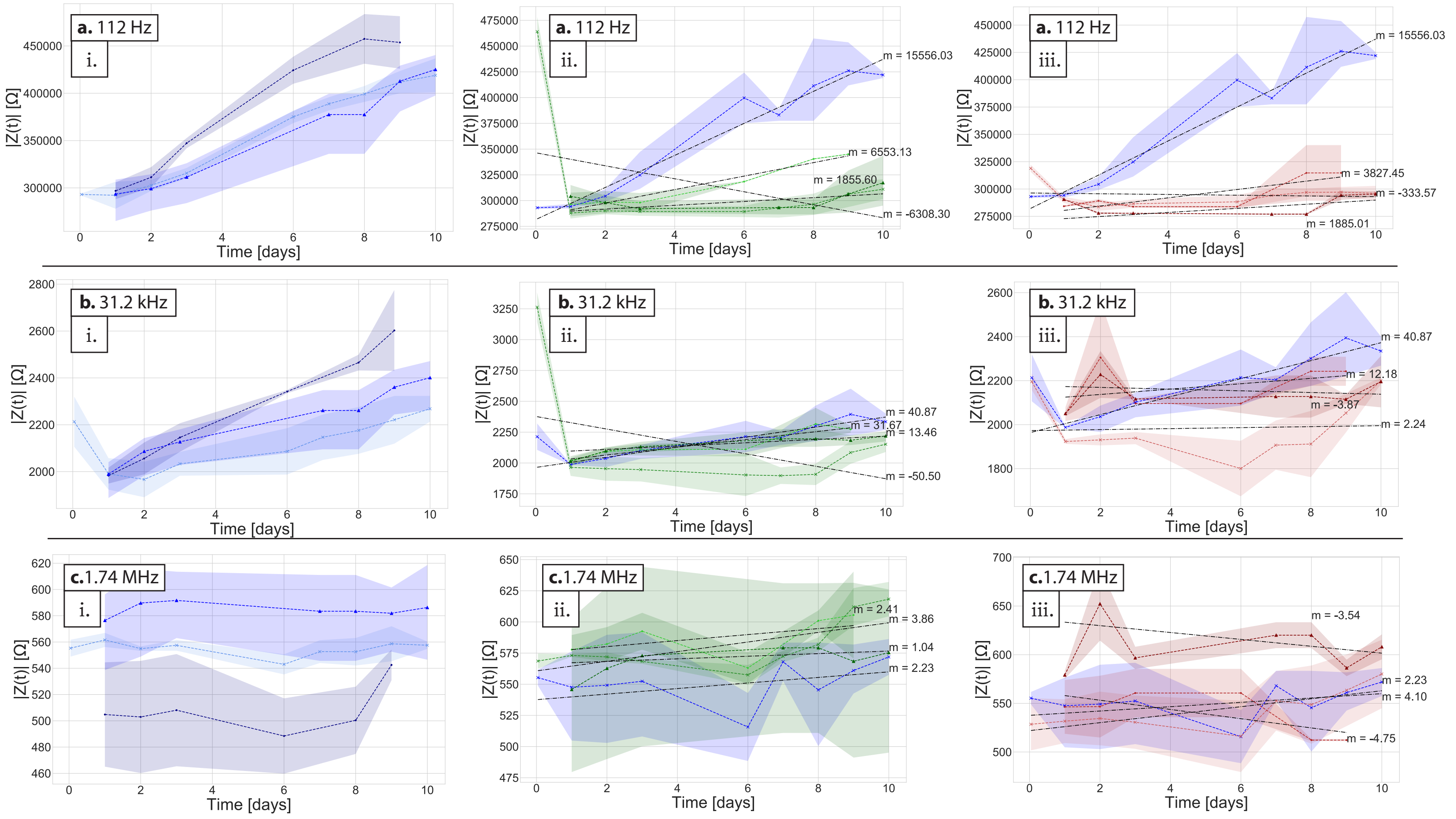

Figure S3. Impedance magnitude plots depicting measurements taken daily at (a) 112 Hz, (b) 31.2 kHz and (c) 1.74 MHz while the electrodes were incubated in standard culture conditions. (a,b,c-i) show the impedance magnitude of the control replicates at the different frequencies, blue lines are from wells containing collagen in the media DMEM + FBS. (a,b,c-ii) show the averaged impedance magnitude of the control replicates in blue line at the different frequencies, green lines are from wells seeded with 3×10^4 cells in collagen as well as the media. (a,b,c-iii) show the averaged impedance magnitude of the control replicates in blue line at the different frequencies, dark red lines are from wells initially seeded with 15×10^5 cells in collagen as well as the media. The shaded area shows the minimum and maximum of the replicates in each experiment and the dashed lines show the slope fitted to each measurement.

Table S1. Elemental data obtained from the X-ray photoelectron spectroscopy (XPS) measurements.

Sample	C 1s	O 1s	Au 4d	Si 2s	N 1s
Bare electrode	48.3	32.3	6.6	12.8	
Bare electrode	49.8	31.3	5.9	13.0	
+ Cystine	62.6	17.2	15.0	5.2	
+ Cystine	65.5	17.0	12.4	5.2	
+ Cystine + Media	61.4	19.7	3.9	4.3	10.7
+ Cystine + Media	63.8	17.9	3.4	4.3	10.6
+ Cystine + Media + Collagen	63.5	20.1	2.8	2.6	11.0
+ Cystine + Media + Collagen	62.3	19.3	5.2	3.0	10.3

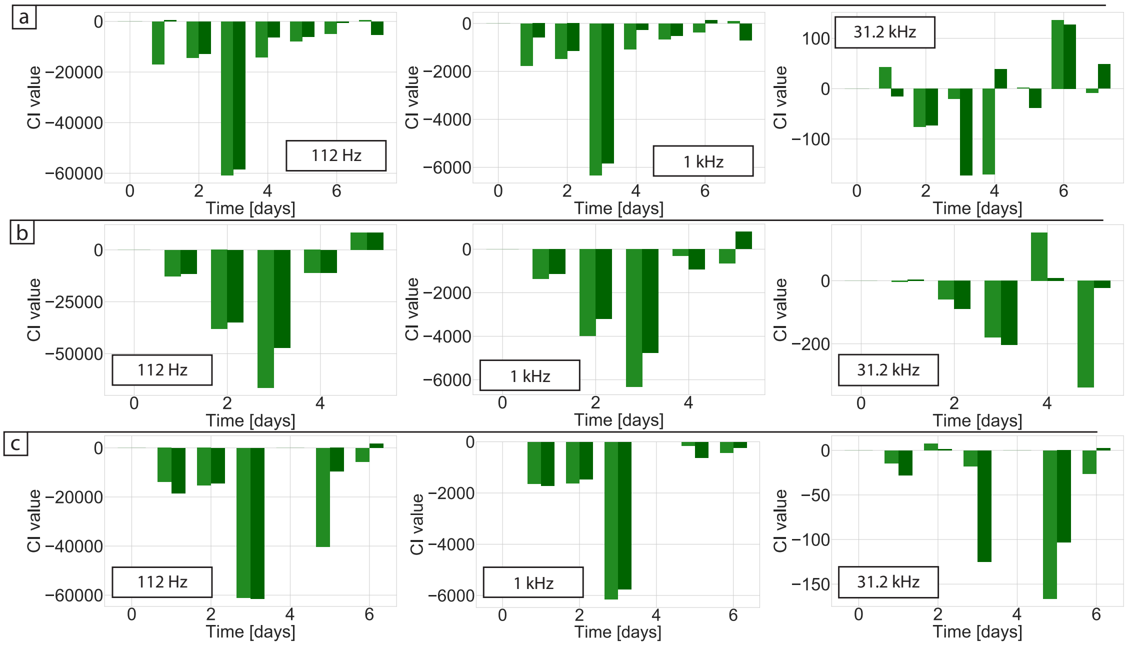

Figure S4. Impedance magnitude plots depicting measurements taken daily at 112 Hz, 31.2 kHz and 1.74 MHz while the electrodes were incubated in standard culture conditions with 3D cultures. Data was normalised using $CI_{(n)} = |I_{cells(n)}| - |I_{cells(n-1)}| - |I_{nocells(n)}| - |I_{nocells(n-1)}|$. (**a**), (**b**), (**c**) are the different biological replicates.
